# Long-term Exposure to PM_2.5_ and Incidence of Acute Myocardial Infarction

**DOI:** 10.1289/ehp.1205284

**Published:** 2012-11-29

**Authors:** Jaime Madrigano, Itai Kloog, Robert Goldberg, Brent A. Coull, Murray A. Mittleman, Joel Schwartz

**Affiliations:** 1 The Earth Institute, and; 2 Mailman School of Public Health, Columbia University, New York, New York, USA; 3 Department of Environmental Health, Harvard School of Public Health, Boston, Massachusetts, USA; 4 University of Massachusetts Medical School, Worcester, Massachusetts, USA; 5 Department of Biostatistics, Harvard School of Public Health, Boston, Massachusetts, USA; 6 Cardiovascular Epidemiology Research Unit, Beth Israel Deaconess Medical Center, Boston, Massachusetts, USA; 7 Department of Epidemiology, Harvard School of Public Health, Boston, Massachusetts, USA

**Keywords:** air pollution.

## Abstract

Background: A number of studies have shown associations between chronic exposure to particulate air pollution and increased mortality, particularly from cardiovascular disease, but fewer studies have examined the association between long-term exposure to fine particulate air pollution and specific cardiovascular events, such as acute myocardial infarction (AMI).

Objective: We examined how long-term exposure to area particulate matter affects the onset of AMI, and we distinguished between area and local pollutants.

Methods: Building on the Worcester Heart Attack Study, an ongoing community-wide investigation examining changes over time in myocardial infarction incidence in greater Worcester, Massachusetts, we conducted a case–control study of 4,467 confirmed cases of AMI diagnosed between 1995 and 2003 and 9,072 matched controls selected from Massachusetts resident lists. We used a prediction model based on satellite aerosol optical depth (AOD) measurements to generate both exposure to particulate matter ≤ 2.5 μm in diameter (PM_2.5_) at the area level (10 × 10 km) and the local level (100 m) based on local land use variables. We then examined the association between area and local particulate pollution and occurrence of AMI.

Results: An interquartile range (IQR) increase in area PM_2.5_ (0.59 μg/m^3^) was associated with a 16% increase in the odds of AMI (95% CI: 1.04, 1.29). An IQR increase in total PM_2.5_ (area + local, 1.05 μg/m^3^) was weakly associated with a 4% increase in the odds of AMI (95% CI: 0.96, 1.11).

Conclusions: Residential exposure to PM_2.5_ may best be represented by a combination of area and local PM_2.5_, and it is important to consider spatial gradients within a single metropolitan area when examining the relationship between particulate matter exposure and cardiovascular events.

Several studies have shown associations between chronic exposure to particulate air pollution and increased mortality, particularly from cardiovascular disease (Dockery et al. 1993; Pope et al. 2004; Puett et al. 2009). Fewer studies, however, have examined the association between long-term exposure to fine particulate air pollution, such as particulate matter ≤ 2.5 μm in diameter (PM_2.5_), and specific cardiovascular outcomes, such as acute myocardial infarction (AMI). A systematic review of the association between air pollution and the incidence of MI concluded that the evidence for long-term effects, in contrast to short-term effects, of air pollution on MI risk is limited and few conclusions could be drawn (Bhaskaran et al. 2009). This may be in part because of the limited number of AMIs in many cohort studies.

Furthermore, spatial gradients within metropolitan areas are increasingly being identified as important in the association between particulate air pollution and health outcomes. Findings suggest that spatial gradients within cities might be as large, or larger, as those between cities (Hoek et al. 2002; Jerrett et al. 2005). In one of the few cohort studies that has investigated long-term exposure to PM_2.5_ and incidence of cardiovascular events, a larger association with AMI was found for an exposure increase of 10 μg/m^3^ within cities than between cities (Miller et al. 2007). The hazard ratio for AMI did not reach statistical significance, but this study had limited statistical power because of the relatively small number (*n* = 584) of events. This limitation is a common problem for even large cohort studies; the incidence of AMI in a decade is not high enough to produce a large number of cases. In such circumstances, case–control studies are an attractive alternative.

We previously found an association between traffic particles and occurrence of AMI in case–control studies within a single metropolitan area (Tonne et al. 2007, 2009). Our earlier analyses included indicators of traffic as a proxy for long-term exposure to traffic pollutants as well as a latent-variable approach to model residential exposure to traffic particles. Hence, it did not capture any effects of particles other than primary traffic particles. To gain a better understanding of how long-term exposure to area particulate matter affects the onset of AMI, and to distinguish between area and local pollutants, we examined both of these measures simultaneously in our analysis. In the present study, we used a PM_2.5_-prediction model based on satellite aerosol optical depth (AOD) measurements (Kloog et al. 2011). The model generates area particulate air pollution predictions in addition to local particulate pollution based on local land use variables, both of which are assigned according to residential address. We then examined the association between both area and local particulate pollution and incidence of AMI using a case–control study design.

## Methods

*Study population.* Cases of AMI included in this study were drawn from the Worcester Heart Attack Study, an ongoing community-wide investigation examining changes over time in the incidence and case-fatality rates of independently confirmed cases of AMI in residents of the greater Worcester, Massachusetts, area who were hospitalized with MI at all area medical centers. The details of this study have been described previously (Floyd et al. 2009; Goldberg et al. 1988, 1999). In brief, during the 5 years under study for the present investigation (1995, 1997, 1999, 2001, and 2003), the medical records of the 11 acute care general hospitals serving residents of the Worcester metropolitan area were searched for patients with a possible discharge diagnosis of AMI. The records were reviewed and validated according to diagnostic criteria described previously (Floyd et al. 2009; Goldberg et al. 1988), and at least two of the following criteria were required for inclusion in the original study: a suggestive clinical history, increased serum biomarker levels above each hospital’s normal range, and serial electrocardiographic findings indicative of AMI. The present investigation was limited to patients ≥ 25 years of age who were hospitalized with independently confirmed AMI.

Population controls were randomly selected from resident lists published in 2003. Resident lists are published annually by each town in Massachusetts and include all residents ≥ 17 years of age. Inclusion in the list is mandated by state law and is based on response to a mailing or visit by the town registrar. Information included in the lists varied from town to town, but at a minimum included name, street address, sex, and year of birth. There were twice as many controls selected for the present investigation as there were cases. Controls were frequency-matched to cases on the basis of age (in 10-year categories), sex, and section of the study area (one of three regions of roughly equal population), such that controls were selected independently of residential location within section. The study area sections were central Worcester, the northern suburbs, and the southern suburbs.

Cases’ residential addresses at the time of AMI were collected from the review of hospital medical records, and the controls’ residential addresses were extracted from the resident lists. Addresses were sent to a commercial firm for geocoding (Mapping Analytics, Rochester, NY). The study was approved by the Committee for the Protection of Human Subjects at the University of Massachusetts Medical School and the Human Subjects Committee at the Harvard School of Public Health and was exempt from informed consent requirements.

*Exposure.* Our long-term exposures of interest were area PM_2.5_ and residual local PM_2.5_ pollution due to traffic. Both long-term area PM_2.5_ and local PM_2.5_ exposure at the residence for the year 2000 were generated by a novel exposure model developed recently by Kloog et al. (2011) for assessing temporally and spatially resolved PM_2.5_ exposures for epidemiological studies. This new method uses MODIS (Moderate Resolution Imaging Spectroradiometer) satellite-derived AOD measurements to predict daily PM_2.5_ concentration levels at a 10 × 10 km spatial resolution in New England beginning in the year 2000 based on daily physical measurements of a surrogate for PM_2.5_ concentrations in each grid cell. In brief, we performed day-specific calibrations of AOD data using ground PM_2.5_ measurements from 78 monitoring stations and land use regression and meteorological variables (temperature, wind speed, visibility, elevation, distance to major roads, percent of open space, point emissions, and area emissions). To estimate PM_2.5_ concentrations in each grid cell on each day we calibrated the AOD–PM_2.5_ relationship for each day using data from grid cells with both monitor and AOD values, using mixed models with random slopes for day and nested regions. In a second stage, we estimated exposures on days when AOD measures were not available (due to cloud coverage or snow, for example). A model was fit with a smooth function of latitude and longitude and a random intercept for each cell (similar to universal kriging) that takes advantage of associations between grid cell AOD values and PM_2.5_ data from monitors located elsewhere and associations with available AOD values in neighboring grid cells.

To validate our model, the data set was repeatedly randomly divided into 90% and 10% splits. Predictions for the held-out 10% of the data were made from the model fit of the remaining 90% of the data. This “out of sample” process was repeated 10 times and cross-validated *R*^2^ values were computed. The first stage calibrations resulted in high out-of-sample 10-fold cross-validated *R*^2^ (mean out-of-sample *R*^2^ = 0.85). Even for location-day combinations without AOD data (the second stage models), our model performance was excellent (mean out-of-sample *R*^2^ = 0.81). Importantly, these *R*^2^ are for daily observations, rather than monthly or yearly values. To check for bias we regressed the measured PM_2.5_ values against the predicted values in each site on each day.

To estimate traffic particle exposures at the local level, we used local (100 m) land use terms (distance to primary highways, distance to point source emissions, population density, percent of open spaces, elevation, and traffic density) to model the difference between the 10 × 10 km grid cell predictions and monitored values. We regressed the residuals for each monitor against local land use characteristics for each monitor and a smooth function of traffic density. The local PM_2.5_ term provides an estimate of traffic-related local particulate pollution that is spatiotemporally correlated with PM_2.5_. Finally, as an estimate of the total outdoor PM_2.5_ exposure at residential location, we summed the local and area PM_2.5_ terms.

*Covariates.* Because our exposure varied spatially, confounding by spatially varying covariates was an issue. We obtained area-based measures of socioeconomic status (SES) from the year 2000 census at the block group level (U.S. Census Bureau 2000b). The following SES measures were obtained: proportion of the population with 1999 income below the federally defined poverty level, median household income in 1999, and percentage of persons ≥ 25 years of age whose highest degree was less than a high school diploma or its equivalent. Census block groups have a population of about 1,500 individuals and are defined by the Census Bureau as small statistical subdivisions of counties with generally stable boundaries, designed to have relatively homogeneous demographic and economic characteristics (U.S. Census Bureau 2000a). Census block group data on poverty have been shown to be a relatively sensitive measure of socioeconomic inequalities in health outcomes (Krieger et al. 2002).

Individual lifestyle factors, such as dietary patterns, obesity, and level of exercise, may be correlated with place of residence. Although such lifestyle factors were unavailable for the cases and controls in our study, we attempted to control for such factors by proxy. Obesity prevalence, as well as fruit and vegetable consumption, have been associated with distance to large supermarkets (defined as having > 50 employees) in metropolitan areas (Michimi and Wimberly 2010), whereas access to parks, walking and jogging trails, and enjoyable scenery have been associated with physical activity behavior (Brownson et al. 2001). ArcGIS version 10.1 (ESRI, Redlands, CA) was used to calculate the straight-line distance between residential addresses and large supermarkets. Locations of large supermarkets were available from the 2006 infoUSA Business Listing File from ESRI’s Business Analyst Extension. Data on recreation areas was downloaded from the MassGIS web site (MassGIS 2012).

*Statistical analysis.* We first ran logistic regression models adjusted only for the matching factors—age, sex, and section of the study area. Although we matched on 10-year age groups, we included age in our models as a continuous, linear term, thus controlling more finely for this covariate. We also included all higher-order (2- and 3-way) interaction terms for the matching factors in our models. Next, we included measures of block group population density and SES, distance to the nearest large supermarket, and distance to nearest recreation area in our models. Finally, we used generalized estimating equations (GEEs) assuming an exchangeable correlation structure within census block group and census tract to account for any remaining correlation among subjects in the same block group (or census tract) not captured by model covariates. We ran two sets of models: the first with separate terms for area and local PM_2.5_, and the second with a term for their sum. In addition, we repeated GEE models restricted to first (vs. any) AMI, and after stratifying by section of the study area, and by time period (1995, 1997, and 1999; or 2001 and 2003). All models were conducted using PROC GENMOD in SAS version 9.2 (SAS Institute Inc., Cary, NC).

## Results

**Figure 1:**
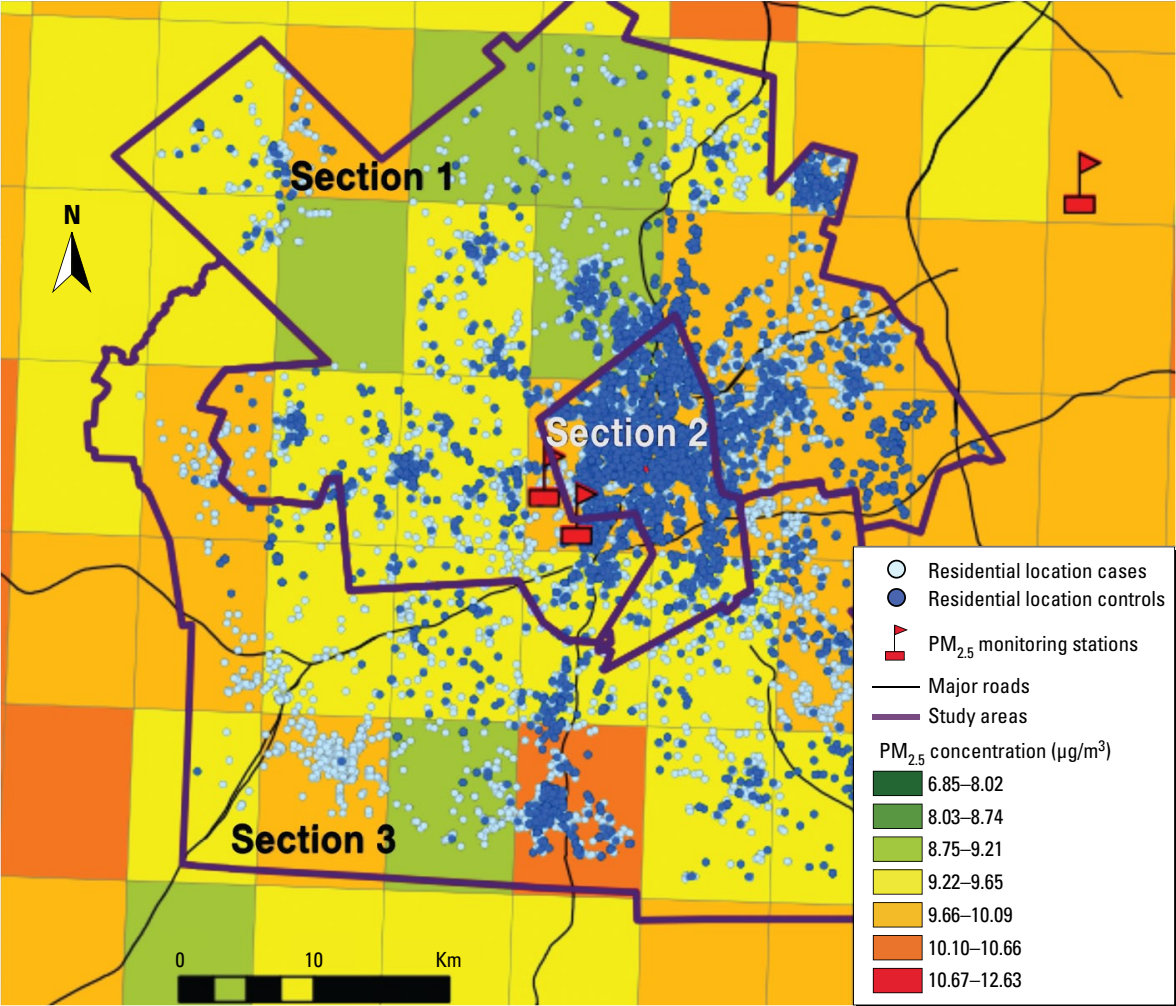
Map of the study area (Worcester, MA) showing the residential location of subjects (cases and controls) with a sample mean PM_2.5_ (µg/m^3^) 10 × 10 km pollution grid for the year 2000.

Exposure and covariate information by study area section for cases and controls is presented in Table 1. Figure 1 shows the residential location for our study subjects according to their 10 × 10 km pollution grid cell. Exposure was divided into two parts: *a*) area PM_2.5_ predicted for the 10 × 10 km grid cell that each case or control lived in, and *b*) local PM_2.5_ from the local land use prediction model. In the year 2000, the interquartile range (IQR) for area PM_2.5_ was 0.6 μg/m^3^, and the IQR for local PM_2.5_ was 1.1 μg/m^3^. Area PM_2.5_ was highest in section 2 of our study area, but there was more variability in this exposure metric in sections 1 and 3 (Table 1). Local PM_2.5_ (and the variability of local PM_2.5_ estimates) was highest in section 1. We also examined the degree to which exposure was correlated with area-based measures of SES in control subjects. There was moderate correlation between area PM_2.5_ and percent poverty within a census block group (ρ = 0.35), but a small inverse correlation with local PM_2.5_ (ρ = –0.07), resulting in a weak correction with total PM_2.5_ (ρ = 0.1).

**Table 1:** Characteristics of cases and controls by study section [mean ± SD or n (%)].

Characteristic	All		Section 1		Section 2		Section 3	
	Cases (n = 4,467)	Controls (n = 9,072)	Cases (n = 1,546)	Controls (n = 3,196)	Cases (n = 2,074)	Controls (n = 4,296)	Cases (n = 847)	Controls (n = 1,580)
Age (years)	70 ± 14	69 ± 14	70 ± 14	69 ± 14	71 ± 14	71 ± 14	68 ± 14	67 ± 15
Male	2,559 (57.3)	5,228 (57.6)	935 (60.5)	1,937 (60.6)	1,130 (54.5)	2,362 (55.0)	494 (58.3)	929 (58.8)
Population density (individuals per km2)	1,848 ± 2,350	1,695 ± 2,178	648 ± 596	655 ± 631	3,250 ± 2,786	2,905 ± 2,606	605 ± 693	509 ± 627
Distance to large supermarket (km)	2.9 ± 2.6	2.8 ± 2.6	4.1 ± 3.4	3.7 ± 3.3	1.6 ± 0.8	1.6 ± 0.8	4.0 ± 2.3	4.5 ± 2.5
Distance to recreation area (m)	532 ± 574	538 ± 523	452 ± 430	470 ± 469	429 ± 302	469 ± 308	932 ± 990	861 ± 862
Block group SES
Median income ($)	48,034 ± 19,201	49,836 ± 18,457	59,460 ± 17,839	59,984 ± 16,864	37,847 ± 16,419	40,868 ± 16,726	52,121 ± 13,960	53,695 ± 13,352
Percent residents living < the poverty line	10.6 ± 11.7	9.1 ± 10.4	4.7 ± 3.8	4.3 ± 3.5	16.5 ± 14.3	13.7 ± 13.0	6.9 ± 5.0	6.3 ± 4.5
Percent residents with < high school education	17.9 ± 12.3	16.4 ± 11.2	10.5 ± 6.7	10.4 ± 6.2	23.7 ± 13.5	21.2 ± 12.7	17.4 ± 9.0	15.7 ± 8.6
Exposure (μg/m3)
Area PM2.5	9.43 ± 0.44	9.39 ± 0.45	9.29 ± 0.51	9.24 ± 0.51	9.58 ± 0.3	9.54 ± 0.33	9.34 ± 0.49	9.30 ± 0.48
Local PM2.5	1.07 ± 1.56	1.04 ± 1.36	1.12 ± 2.36	1.07 ± 1.95	1.03 ± 0.85	1.04 ± 0.85	1.08 ± 0.93	1.01 ± 0.94
Total PM2.5	10.50 ± 1.55	10.44 ± 1.36	10.41 ± 2.37	10.31 ± 1.97	10.6 ± 0.79	10.58 ± 0.8	10.42 ± 0.96	10.3 ± 0.96

In our models adjusted only for matching factors, exposure to area PM_2.5_ was associated with occurrence of AMI. This association was robust to additional adjustment for population density, SES, distance to a large supermarket, and distance to a recreation area, although slightly attenuated (Table 2). An IQR increase in area PM_2.5_ (0.59 μg/m^3^) was associated with a 16% increase in the odds of AMI (95% CI: 1.04, 1.29). IQR increases in local PM_2.5_ (1.08 μg/m^3^) and total PM_2.5_ (1.05 μg/m^3^) were weakly associated with occurrence of AMI after adjusting for area PM_2.5_ and other covariates (Table 2). Estimates from models with an exchangeable correlation structure for census block group or tract were not materially different. Restricting the outcome to first (vs. any) AMI had little influence on associations; however, estimates varied somewhat among the three study area sections and when stratified according to time period (Table 3).

**Table 2:** Relative odds [OR (95% CI)] of AMI among cases and controls.

Model	Area PM2.5		Local PM2.5		Total PM2.5	
Area PM2.5 onlya	1.15	(1.09, 1.21)	—	—	
Area PM2.5 and local PM2.5a	1.16	(1.10, 1.22)	1.03	(1.00, 1.06)	1.04	(1.01, 1.08)
Area PM2.5 and local PM2.5b	1.12	(1.06, 1.18)	1.03	(1.00, 1.07)	1.04	(1.00, 1.07)
GEE models with exchangeable correlation within census block groupb	1.16	(1.04, 1.29)	1.03	(0.97, 1.10)	1.04	(0.96, 1.11)
GEE models with exchangeable correlation within census tractb	1.18	(1.04, 1.35)	1.03	(0.98, 1.09)	1.03	(0.97, 1.10)
OR per IQR of pollutant; the IQR was 0.59 μg/m3 for area PM2.5, 1.08 μg/m3 for local PM2.5, and 1.05 μg/m3 for total PM2.5. aAdjusted for matching factors (age, sex, and study area section) and interaction terms of matching factors. bAdjusted for matching factors (age, sex, and study area section), interaction terms of matching factors, population density, SES, distance to large supermarket, and distance to recreation area.						

**Table 3:** Relative odds [OR (95% CI)] of AMI among cases and controls.

Stratification factor	Area PM2.5		Local PM2.5		Total PM2.5	
AMI order
First AMI	1.19	(1.06, 1.33)	1.04	(1.00, 1.09)	1.05	(1.00, 1.11)
Any AMI	1.16	(1.04, 1.29)	1.03	(0.97, 1.10)	1.04	(0.97, 1.11)
Study section
1	1.24	(1.04, 1.48)	1.01	(0.96, 1.06)	1.01	(0.95, 1.07)
2	1.18	(1.04, 1.35)	1.06	(0.98, 1.14)	1.06	(0.98, 1.13)
3	1.06	(0.83, 1.34)	1.11	(0.99, 1.24)	1.10	(0.99, 1.23)
Study period
1995, 1997, 1999	1.10	(0.98, 1.24)	1.03	(0.98, 1.08)	1.03	(0.98, 1.09)
2001, 2003	1.18	(1.05, 1.32)	1.00	(0.96, 1.03)	1.00	(0.96, 1.05)
OR per IQR of pollutant; the IQR was 0.59 μg/m3 for area PM2.5, 1.08 μg/m3 for local PM2.5, and 1.05 μg/m3 for total PM2.5. GEE models were used with exchangeable correlation within census block group; adjusted for matching factors (age, sex, and study area section), interaction terms of matching factors, population density, SES, distance to large supermarket, and distance to recreation area.						

## Discussion

In the present analysis, we observed an association between long-term exposure to area PM_2.5_, a regional air pollutant, and occurrence of AMI. Although several studies have found associations between long-term exposure to PM_10_ or PM_2.5_ and cardiovascular disease mortality (Dockery et al. 1993; Krewski et al. 2009; Miller et al. 2007; Puett et al. 2009), few have looked at specific outcomes such as AMI. In two prospective studies of women, one across the United States and one in the Northeast and Midwest regions of the country, elevated, but not statistically significant, hazard ratios were found for incident MI in association with an increase of 10 μg/m^3^ of PM_2.5_, with exposure based on either nearest monitor (Miller et al. 2007) or a spatiotemporal regression model (Puett et al. 2009). The relatively small number of incident cases in the two studies (< 1,000 in each) may partly explain these findings. In contrast, the present study included > 4,000 incident cases of AMI.

Previous analyses (Tonne et al. 2007, 2009) of the Worcester Heart Attack Study, a population-based case–control study, indicated that exposure to traffic particles was associated with occurrence of AMI. In the present study, we found an association between the occurrence of AMI and exposure to regional PM_2.5_ while controlling for fine-scale variation in particulate air pollution that may be due to local traffic. We modeled exposure of PM_2.5_ based on daily measurements of AOD in 32 grid cells across Worcester County. The use of actual spatially resolved measurements is an important advantage over land use regression (LUR), which is calibrated using space- and time-limited monitoring data, and our model performed better in out-of-sample validation than reported previously in other LUR-based models (Kloog et al. 2011). LUR models are calibrated only at measuring sites; our model benefits by incorporating physical measurements (via satellite data) over the entire spatial domain. In addition, satellite AOD data may be used to fit LUR models in locations without ground monitors, and may reduce bias due to non-random placement of monitors.

We separated estimates for local- and area-level pollution in our modeling. The first phase used the model developed by Kloog et al. (2011) to estimate average PM_2.5_ concentrations on a 10 km grid. In the second phase, we took the residuals between the actual monitored value in each grid and the predicted mean value for each grid, which presumably reflect the influence of local conditions near each monitoring site, and regressed them against land use terms within 100 m of the monitor to account for the effects of these local sources. This model was then used to estimate local particle concentrations at the addresses of study participants. Because the land use regression is fit to the difference between the monitored value and the grid cell prediction, this local contribution is independent of the grid cell value, allowing us to examine the different sources of particle exposure with less collinearity in our model. A unique advantage of this approach is that it allows us to look at these exposure metrics separately and together, allowing for a best estimate of a subject’s residential outdoor PM_2.5_ exposure. Because the measure of total PM_2.5_ comprised two estimates with different spatial variability, it had little correlation with area SES characteristics, such as percent families living in poverty, and therefore the association between this metric and AMI may suffer less from residual confounding.

In the northeastern United States, PM_2.5_ is composed predominantly of secondary organics and sulfate aerosols. Sulfate aerosols are formed from the oxidation of sulfur dioxide (SO_2_) emitted from fossil fuel combustion, and it is estimated that 70% of the SO_2_ emissions in the United States are from electricity-generating facilities (U.S. Environmental Protection Agency 2009). Formation of secondary organic aerosols is not as fully understood as that of particulate sulfate, but a portion of their formation is attributed to aromatic hydrocarbon precursors under nitrogen oxide (NO_X_)-limiting conditions, and NO_X_ is, like SO_2_, also emitted from fossil fuel combustion, including motor vehicle exhaust. Prior work in this cohort was focused on traffic-related air pollution, which was measured using exposure proxies and a latent variable model (Tonne et al. 2007, 2009). However, in the present analysis we were able to estimate personal exposure to total PM_2.5_, which was not solely due to traffic. A key contribution of the present analysis is the finding that transported particles, as well as local traffic particles, are associated with cardiovascular disease.

Through the use of our spatiotemporal regression model, we also identified an association between AMI and a relatively small amount of variation in PM_2.5_ exposure within a single New England metropolitan area over the course of a calendar year. These results are consistent with findings from the Women’s Health Initiative Observational Study, where the association between PM_2.5_ and cardiovascular events was stronger within-cities than between-cities (Miller et al. 2007). Taken together, these findings indicate that it is important to examine variation in exposure within a single metropolitan area, even when examining regional air pollutants, such as PM_2.5_. Our results were not attenuated when accounting for spatial dependence, possibly because of the varying spatial scales of our exposure metrics. As expected, local sources made the greatest contribution to variation in particulate matter exposure, and therefore local PM_2.5_ accounted for most of the variation in total PM_2.5_ in this single metropolitan area. However, there was enough variation in area PM_2.5_ to detect an independent association with that exposure metric as well.

Fine control for socioeconomic factors at the block group level, which in urban areas is quite small, had little effect on associations with PM_2.5_. This is consistent with a recent publication of Brochu et al. (2011), who showed that PM_2.5_ concentrations were associated with measures of poverty, education, and income over long spatial scales representing regional and between-city differences, but not on the finer within-city spatial scale, suggesting that studies focusing on within-city spatial variation will have little confounding with SES measures. Indeed, we found low-to-moderate correlation between our exposure metrics and measures of socioeconomic characteristics at the population level.

In contrast to our previous analysis specifically examining traffic particles, we only found a weak association between our measure of local PM_2.5_ pollution and occurrence of AMI. Our estimate of “residual” local variation in particulate matter can be thought of as the incremental effect, beyond that captured by area PM_2.5_, of particulate air pollution. The fact that it represents only an incremental effect, or that it captures a different source of pollution, may explain this difference.

A number of mechanisms by which long-term exposure to PM_2.5_ may impact cardiovascular disease have been proposed, such as progression of atherosclerosis, systemic inflammation, and alterations in immune function. Evidence for such mechanistic pathways exists in both the toxicology and epidemiology literature. Studies of apolipoprotein E–deficient (ApoE^–/–^) mice have linked exposure to concentrated air particles over 4–6 months with increased aortic atherosclerotic plaque (Chen and Nadziejko 2005). A more recent study of low density lipoprotein receptor–deficient (LDLR^–/–^) mice demonstrated that particle exposure increased oxidation of LDL, increased the thickness of the arterial wall, and promoted plaque growth and instability (Soares et al. 2009). In humans, long-term exposure to PM_2.5_ has been associated with increased carotid intima media thickness, a subclinical marker of coronary atherosclerosis, in two cross-sectional studies in the United States (Diez Roux et al. 2008; Künzli et al. 2005) and one in Germany (Bauer et al. 2010). Other studies have also reported associations of particles with various markers of chronic atherosclerosis (Adar et al. 2010; Allen et al. 2009). These studies suggest that our findings of an association between long-term exposure to PM_2.5_ and occurrence of AMI are biologically plausible.

In the present population-based study, we observed an association between AMI and PM_2.5_ exposure. However, this study is not without limitations, and therefore these findings should be interpreted with caution. Because our exposure varied spatially, we included other spatially varying covariates that also predicted AMI, such as percent of households living in poverty, distance to large supermarkets, and distance to recreation areas, in our models. However, these measures do not perfectly account for individual-level AMI risk factors (e.g., smoking, dietary patterns, physical activity) that also vary spatially, and therefore could be a source of unmeasured confounding in our models. We attempted to account for this by running models that included an exchangeable correlation structure within census block groups and census tracts, which did not change results substantially. Nonetheless, some residual confounding by socioeconomic and lifestyle factors is likely. Associations varied, somewhat, by section of the study area, which may be a function of varying exposure and residual confounding.

Our PM_2.5_ prediction models have a relatively coarse spatial resolution (10 × 10 km), which may have led to some error in characterizing area-level exposure. Although estimation conducted at a finer spatial resolution is preferable, the ability to capture background area PM_2.5_ and still account for local PM_2.5_ by the separate covariate was an advantage of this study. Models used to predict exposures were also limited by a lack of data on the exact composition of AOD particles. In addition, our model predicted ambient PM_2.5_ exposure at a subject’s residential location, without accounting for the amount of time spent in other locations, indoors versus outdoors, or the length of residence at the current address. Finally, our area PM_2.5_ exposure metric was approximated from the year 2000 annual exposure for the study area. This year was selected based on AOD data availability and because it was within the study period of case accrual. Because our cases were accrued before and after this date, we do not expect differential exposure error in the cases, but it is possible that there is some differential exposure misclassification with respect to residential history for the controls. When we stratified by study time period, the OR for the area PM_2.5_ estimate was greater during the later time period. Because controls were sampled by proxy from resident lists in 2003 to represent the study base over the entire study period, exposure estimates for controls in the earlier time periods may have been higher than the actual exposure for these subjects, leading to a downward bias of our results.

## Conclusions

After accounting for local pollution exposure, long-term exposure to area PM_2.5_ was associated with the occurrence of AMI in this population-based study. The association between total PM_2.5_ and AMI occurrence was weaker, but this metric of combined spatial scales may provide a better estimate of total PM_2.5_ exposure at an individual’s residence. This study adds to the growing body of literature on long-term regional particulate pollution and cardiovascular morbidity, and highlights the importance of examining pollutant variability within a single metropolitan area, rather than solely focusing on comparisons across large spatial scales.
